# Isolated Renal Metastasis from Primary Lung Squamous Cell Carcinoma with Synchronous Small Cell Lung Cancer

**DOI:** 10.7759/cureus.4891

**Published:** 2019-06-12

**Authors:** Laith Numan, Samia Asif, Omar K Abughanimeh

**Affiliations:** 1 Internal Medicine, University of Missouri-Kansas City School of Medicine, Kansas City, USA; 2 Hematology/Oncology, University of Nebraska Medical Center, Omaha, USA

**Keywords:** lung mass, synchronous lung cancer, cancer, metastasis, kidney metastasis

## Abstract

Synchronous multiple primary lung cancer is a unique type of lung carcinomas that are diagnosed with more than two different pathological types in the same or different lung lobes. Isolated metastasis to the kidney is considered rare. Herein, we present a case of a 58-year-old male with a history of chronic obstructive pulmonary disease (COPD) and 40 pack-year of cigarette smoking, who was diagnosed with synchronous small cell lung cancer (SCLC) and squamous cell carcinoma (SCC) with isolated metastasis to the kidney. Isolated kidney metastasis from lung cancer is an infrequent finding; it should be considered when the patient is diagnosed with lung cancer. In the absence of disseminated disease and contraindications, nephrectomy is an option for treatment with chemotherapy or as a palliative measure if the patient is symptomatic.

## Introduction

Synchronous occurrence of primary lung cancers with different histology is uncommon, seen in up to only 0.5% of patients with lung cancer [1-2]. Non-small cell lung cancer (NSCLC) constitutes 80% of all lung cancers, and squamous cell carcinoma (SCC) accounts for 40% of these [3]. SCC remains the most common type of lung malignancy seen in any combination of synchronous lung tumors [1]. High mortality resulting from lung cancer has been attributed to metastatic involvement of distant organs. Frequent sites of metastasis include bone, brain, and liver [4]. Isolated kidney involvement by primary lung cancer is a rare entity.

Herein, we present an unusual case where a patient was diagnosed with concurrent primary lung SCC and SCLC and was subsequently discovered to have isolated unilateral renal metastasis.

## Case presentation

**Figure 1:**
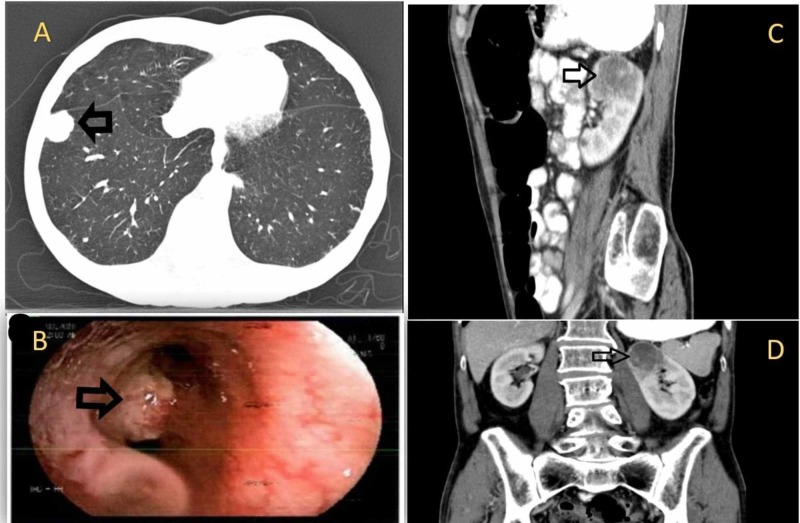
A: CT scan showing the right lower lobe nodule, B: Bronchoscopy image showing the left main bronchus mass, C & D: CT scan showing the isolated left kidney metastasis CT, computed tomography

A 58-year-old male with a past medical history of coronary artery disease, chronic obstructive pulmonary disease (COPD), and 40 pack-year history of cigarette smoking presented to the emergency department complaining of shortness of breath and chest pain. He was afebrile, tachypneic, tachycardic, and normotensive. On physical examination, he looked cachectic and in mild distress. The patient had decreased air entry on the right lung field; no wheezing or crackles were appreciated. On further examination, he was found to have clubbing of the fingers. His laboratory workup was fairly unremarkable. Chest X-ray showed a nodule in the right lower lung field and left hilar opacity. CT of the chest showed new right lower lobe solid nodule (Figure 1A) and nonspecific infiltrate in the left lower lobe. Pulmonology was consulted, and they recommended CT-guided biopsy for the right lower lobe nodule and bronchoscopy for the left lower lobe infiltrate. Bronchoscopy showed necrotic mass at the left main stem (Figure 1B). Surprisingly, the pathology came back positive for two lung cancer primaries. The right lower lobe nodule came back positive for poorly differentiated carcinoma that favors SCLC. The left lower lobe mass pathology came back positive for invasive keratinizing SCC. The patient underwent staging, including CT abdomen and pelvis, bone scan, and MRI of the brain; he was found to have an isolated mass in the left kidney (Figures 1C, 1D).

Biopsy of the lesion showed metastatic SCC of the lung. Immunohistochemistry ruled out urothelial carcinoma. Urology recommended avoiding any intervention as the renal mass was asymptomatic at this time and to proceed with chemotherapy. The patient was discharged to follow-up with oncology, which later started him on palliative carboplatin/etoposide. He was seen after he finished two cycles of chemotherapy, the patient was ill-looking, and his condition deteriorated. After a lengthy discussion with the patient and his family, he decided to stop chemotherapy and to stay home under the care of the palliative team and his family.

## Discussion

Synchronous lung tumors are defined as two or more tumors that are detected simultaneously but are physically distinct and separate. These may be intra-pulmonary metastases from a primary tumor or two or more concurrently present primary tumors. A retrospective, multi-center study by Rostad et al. evaluated 15,308 patients diagnosed with primary lung cancer over an eight-year study period [5]. Of these, synchronous lung tumors were detected in 94 out of the 2528 patients who underwent surgical resection; only nine patients had primary lung tumors with different histology [5]. This study concluded that compared to patients with distant metastases, patients who had synchronous tumors had a more favorable long-term prognosis if they underwent surgical resection. Compared to synchronous tumors with similar histology, patients with tumors of different histology had more reduced long-term survival, though the number of patients with the latter was small.

The most common sites for lung cancer metastases include the bone, brain, liver, and adrenal glands [4,6]. Prior studies have demonstrated that based on the histological subtype, sites of tumor metastasis may vary. SCLC may show frequent spread to the nervous system and liver; lung adenocarcinoma has a greater propensity for bone and respiratory system involvement. It has been suggested that squamous cell histology is associated with lesser distant metastasis compared to other subtypes [7].

Multiple, bilateral renal metastases and renal deposits in the setting of widespread metastatic disease involving other organs have been reported more commonly [8]. However, isolated renal metastasis is rare; prior reported cases demonstrated that the primary organ of malignancy in these cases is lung, colon/rectum, breast, soft tissue, and thyroid in order of decreasing frequency [9]. A study by Adamy et al. evaluated 3472 patients undergoing partial or total nephrectomy over 20 years; of these, only 13 patients underwent surgery for solitary renal metastasis; five (38%) of these were pulmonary in origin [8].

Patients with tumor spread to the kidneys are usually asymptomatic, being diagnosed incidentally on imaging. Rarely, they may present with gross hematuria, dull abdominal, or back pain or early satiety [3,10]. Diagnosis of renal metastasis is usually by imaging modalities. This includes CT or MRI of the abdomen, positron emission tomography (PET)/CT scan and ultrasonography (US). While differentiating between a renal cortical tumor and a renal metastatic deposit may be difficult based on imaging alone, features supporting the latter diagnosis include the presence of multifocal, endophytic lesions that are isodense or of lower attenuation compared to renal parenchyma and enhance only slightly with intravenous contrast [11-12].

Once diagnosed, no clear guidelines are available for the management of isolated renal involvement. Treatment plans are based on patient outcomes in reported cases in the literature. Primarily, systemic chemotherapy is the mainstay of treatment. Adamy et al. evaluated the role of nephrectomy in the management of these cases and suggested that nephrectomy may be associated with improved survival [8]. An alternative therapeutic option suggested by Verma et al. described the utilization of stereotactic body radiation therapy (SBRT) in the treatment of symptomatic solitary renal deposits where NSCLC was the primary tumor [10]. This was noted to provide adequate local control and relief of presenting symptoms and did not result in any significant adverse effects. No randomized control trials are available to determine the superiority of one treatment modality over the other. Therefore, treatment needs to be individualized for each patient.

## Conclusions

It is anticipated that as survival rates for patients with cancer increase, a higher number of patients will be diagnosed with unusual presentations or rare sites of metastases, such as in our case. The presence of an isolated renal mass in the absence of disease involvement of other organs should not prevent clinical suspicion of kidney involvement by a metastatic process. More studies are needed to determine the best treatment options in cases of isolated renal metastases.

## Appendices

This case was presented as a poster at the American Thoracic Society annual meeting (Isolated Renal Metastasis from Primary Lung Squamous Cell Carcinoma with Synchronous Small Cell Lung Cancer. L Numan, et al. - D61. Thoracic Oncology Case Reports I, 2019. A6924-A6924) 

## References

[1] Yamamoto Y, Kodama K, Yamato H, Takeda M, Takamori H, Karasuno T (2015). Synchronous primary lung cancer presenting with small cell carcinoma and adenocarcinoma. Ann Thorac Cardiovasc Surg.

[2] Hiraki A, Ueoka H, Yoshino T (1999). Synchronous primary lung cancer presenting with small cell carcinoma and non-small cell carcinoma: diagnosis and treatment. Oncol Rep.

[3] Wang J, Wang L, Long L, Tao Q, Xu F, Luo F (2019). Solitary renal metastasis from squamous cell carcinoma of the lung: a case report. Medicine (Baltimore).

[4] Hess KR, Varadhachary GR, Taylor SH (2006). Metastatic patterns in adenocarcinoma. Cancer.

[5] Rostad H, Strand T-E, Naalsund A, Norstein J (2008). Resected synchronous primary malignant lung tumors: a population-based study. Ann Thorac Surg.

[6] Quint LE, Tummala S, Brisson LJ (1996). Distribution of distant metastases from newly diagnosed non-small cell lung cancer. Ann Thorac Surg.

[7] Riihimäki M, Hemminki A, Fallah M (2014). Metastatic sites and survival in lung cancer. Lung Cancer.

[8] Adamy A, Von Bodman C, Ghoneim T, Favaretto RL, Bernstein M, Russo P (2011). Solitary, isolated metastatic disease to the kidney: Memorial Sloan-Kettering Cancer Center experience. BJU Int.

[9] Zhou C, Urbauer DL, Fellman BM (2016). Metastases to the kidney: a comprehensive analysis of 151 patients from a tertiary referral centre. BJU Int.

[10] Verma V, Simone II CB (2017). Stereotactic body radiation therapy for metastases to the kidney in patients with non-small cell lung cancer: a new treatment paradigm for durable palliation. Ann Palliat Med.

[11] Bailey JE, Roubidoux MA, Dunnick NR (2019). Secondary renal neoplasms. Abdom Imaging.

[12] Honda H, Coffman CE, Berbaum KS, Barloon TJ, Masuda K (1992). CT analysis of metastatic neoplasms of the kidney. Acta Radiol.

